# Triphenyl[(triphenylphosphoranylidene)amino]phosphonium tetra­kis­(penta­fluoro­phen­yl)borate

**DOI:** 10.1107/S1600536812049914

**Published:** 2012-12-15

**Authors:** Petr Vanýsek, Chong Zheng

**Affiliations:** a Department of Chemistry and Biochemistry, Northern Illinois University, DeKalb, IL 60115, USA

## Abstract

In the title molecular salt, C_36_H_30_NP_2_^+^·C_24_BF_20_^−^, the P—N bond lengths in the cation are equal [1.573 (2) and 1.572 (2) Å], indicating a resonance structure and the P—N—P bond angle is 144.79 (12)°. In the crystal, weak C—H⋯F interactions link the cations and the anions.

## Related literature

For details of the preparation, see: Fermín *et al.* (1999 ▶); Gobry (2001 ▶). For electrochemical studies of inter­faces between polar organic solvents and water, see: Luo *et al.* (2006 ▶); Fermín *et al.* (1999 ▶); Su *et al.* (2008*a* ▶,*b* ▶); Stephenson *et al.* (2005 ▶). For an X-ray reflectivity study of the inter­face, see: Luo *et al.* (2006 ▶). For a Gibbs free-energy study of the compound, see: Vanýsek & Novák (2009 ▶).
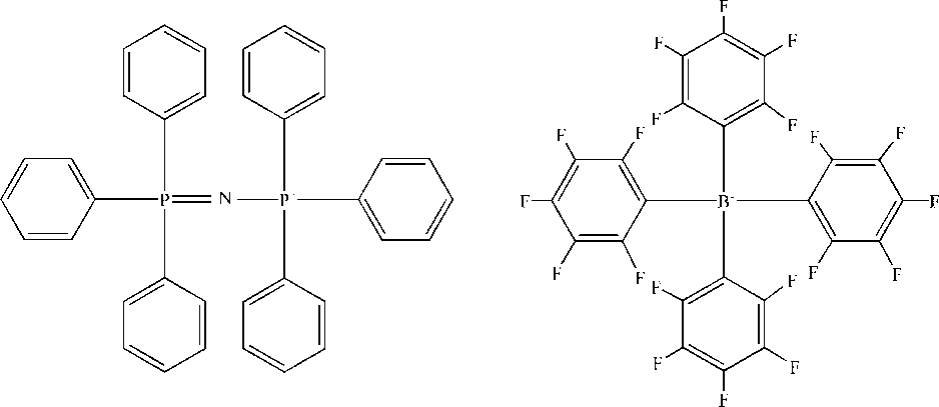


## Experimental

### 

#### Crystal data

C_36_H_30_NP_2_^+^·C_24_BF_20_^−^
*M* *_r_* = 1217.60
Monoclinic, 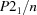

*a* = 13.3081 (15) Å
*b* = 25.196 (3) Å
*c* = 16.0355 (18) Å
β = 100.094 (2)°
*V* = 5293.7 (10) Å^3^
*Z* = 4
Mo *K*α radiation
μ = 0.20 mm^−1^
*T* = 298 K
0.60 × 0.50 × 0.30 mm

#### Data collection

Bruker SMART CCD PLATFORM diffractometer
Absorption correction: multi-scan (*SADABS*; Sheldrick, 2006 ▶) *T*_min_ = 0.212, *T*_max_ = 0.264
39430 measured reflections
9311 independent reflections
7829 reflections with *I* > 2σ(*I*)
*R*_int_ = 0.030

#### Refinement

*R*[*F* ^2^ > 2σ(*F* ^2^)] = 0.038
*wR*(*F* ^2^) = 0.104
*S* = 1.07
9311 reflections
758 parameters
H-atom parameters constrained
Δρ_max_ = 0.28 e Å^−3^
Δρ_min_ = −0.26 e Å^−3^

### 

Data collection: *SMART* (Bruker, 1999 ▶); cell refinement: *SMART* and *SAINT* (Bruker, 1999 ▶); data reduction: *SAINT*; program(s) used to solve structure: *SIR97* (Altomare *et al.*, 1999 ▶); program(s) used to refine structure: *SHELXTL* (Sheldrick, 2008 ▶); molecular graphics: *SHELXTL*; software used to prepare material for publication: *SHELXTL*.

## Supplementary Material

Crystal structure: contains datablock(s) global, I. DOI: 10.1107/S1600536812049914/br2211sup1.cif

Structure factors: contains datablock(s) I. DOI: 10.1107/S1600536812049914/br2211Isup2.hkl

Additional supplementary materials:  crystallographic information; 3D view; checkCIF report

## Figures and Tables

**Table 1:** Hydrogen-bond geometry (Å, °)

*D*—H⋯*A*	*D*—H	H⋯*A*	*D*⋯*A*	*D*—H⋯*A*
C118—H118⋯F302^i^	0.93	2.55	3.188 (2)	126
C212—H212⋯F303^i^	0.93	2.55	3.229 (3)	131

Symmetry code: (i) 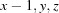.

**Fig. 1.**
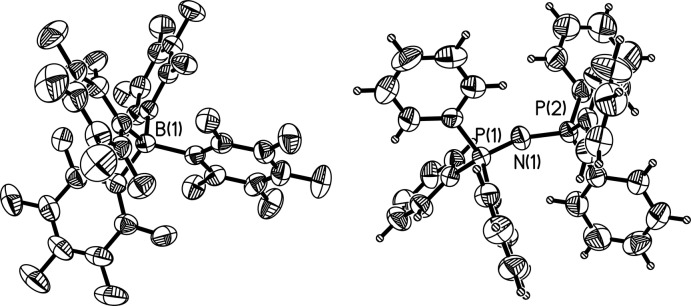
Thermal ellipsoid drawing of the title compound shown at the 50% probability level.

## supplementary crystallographic information

### Comment

Salts of very hydrophobic cations and anions are very desirable for electrochemical studies of interfaces between polar organic solvent and water, commonly known as ITIES (Interface between Two Immiscible Electrolyte Solutions). These systems require an aqueous phase with very hydrophilic salt and a nonaqueous one with very hydrophobic salt, to be used as supporting electrolytes for their respective phases without leaching into the opposite phases. Thus, compound such as the current bis(triphenylphosphoranylidene)-ammonium tetrakis(pentafluorophenyl)borate are natural choice of materials for this purpose (Luo *et al.* 2006, Fermín *et al.* 1999, Su *et al.* 2008*a*,*b*, Stephenson *et al.* 2005). Its CAS registry number is 227603–93-2.

In the work of Luo and coworkers (Luo *et al.* 2006) this compound was used to study the fine structure of interface between two liquids using X-ray reflectivity of the interface. Vanýsek & Novák (2009) calculated the Gibbs energies of the transport for the individual ions between water and dichloroethane, the common organic phase for ITIES work. The structural information is important for understanding these Gibbs free energies.

### Experimental

The preparation method is based on the metathesis of the starting materials with the elimination of water-soluble LiCl. The framework of the procedure is described in (Fermín *et al.* 1999). The starting materials used were bis(triphenylphosphoranylidene)-ammonium chloride (Aldrich) and either lithium or potassium tetrakis(pentafluorophenyl)borate (Boulder Scientific Company). In later preparations potassium tetrakis(pentafluorophenyl)borate (also from Boulder Scientific Company) was used, with identical electrochemical results. Both starting materials were dissolved in a methanol:water 2:1 mixture, with a minimum of 10 ml per gram of starting materials used. The solutions were combined and the precipitate formed was rinsed in copious amounts of a methanol:water 2:1 mixture, followed by large amount of distilled water. The product was vacuum-filtered and dried. The recrystallization was done from hot acetone (Gobry 2001). The yield was 82%. It is possible to obtain higher yields, however, the intended use of the product is very sensitive to any impurity, and the product would deteriorate with higher recovery. Also, it was noted that the vacuum filtration needs to be done rapidly, as prolonged drying on the filter apparently contaminates the product with contaminants from the air, which are visible in the electrochemical work. The melting point of the carefully prepared product was 234–235°C.

### Refinement

The hydrogen atoms on carbon atoms were refined using the riding model in *SHELXL* with the *U*_iso_ equal to 1.5 times of that of the preceding carbon atoms for the methyl groups and 1.3 times for the rings. The C—H distances are equal to 0.97 and 0.96 Å for the CH_2_ and CH_3_ groups, respectively.

### Crystal data

**Table:**

C_36_H_30_NP_2_^+^·C_24_BF_20_^−^	*F*(000) = 2448
*M**_r_* = 1217.60	*D*_x_ = 1.528 Mg m^−^^3^
Monoclinic, *P*2_1_/*n*	Mo *K*α radiation, λ = 0.71073 Å
Hall symbol: -P 2yn	Cell parameters from 264 reflections
*a* = 13.3081 (15) Å	θ = 3–14°
*b* = 25.196 (3) Å	µ = 0.20 mm^−^^1^
*c* = 16.0355 (18) Å	*T* = 298 K
β = 100.094 (2)°	Fragment, colorless
*V* = 5293.7 (10) Å^3^	0.60 × 0.50 × 0.30 mm
*Z* = 4	

### Data collection

**Table:**

Bruker SMART CCD PLATFORM diffractometer	9311 independent reflections
Radiation source: fine-focus sealed tube	7829 reflections with *I* > 2σ(*I*)
Graphite monochromator	*R*_int_ = 0.030
Detector resolution: 0 pixels mm^-1^	θ_max_ = 25.0°, θ_min_ = 1.5°
ω scans	*h* = −15→15
Absorption correction: multi-scan (*SADABS*; Sheldrick, 2006)	*k* = −29→29
*T*_min_ = 0.212, *T*_max_ = 0.264	*l* = −19→19
39430 measured reflections	

### Refinement

**Table:**

Refinement on *F*^2^	Secondary atom site location: difference Fourier map
Least-squares matrix: full	Hydrogen site location: inferred from neighbouring sites
*R*[*F*^2^ > 2σ(*F*^2^)] = 0.038	H-atom parameters constrained
*wR*(*F*^2^) = 0.104	*w* = 1/[σ^2^(*F*_o_^2^) + (0.0481*P*)^2^ + 1.7949*P*] where *P* = (*F*_o_^2^ + 2*F*_c_^2^)/3
*S* = 1.07	(Δ/σ)_max_ = 0.001
9311 reflections	Δρ_max_ = 0.28 e Å^−^^3^
758 parameters	Δρ_min_ = −0.26 e Å^−^^3^
0 restraints	Extinction correction: *SHELXTL* (Sheldrick, 2008), Fc^*^=kFc[1+0.001xFc^2^λ^3^/sin(2θ)]^-1/4^
Primary atom site location: structure-invariant direct methods	Extinction coefficient: 0.0087 (4)

### Special details

**Table:**

Geometry. All e.s.d.'s (except the e.s.d. in the dihedral angle between two l.s. planes) are estimated using the full covariance matrix. The cell e.s.d.'s are taken into account individually in the estimation of e.s.d.'s in distances, angles and torsion angles; correlations between e.s.d.'s in cell parameters are only used when they are defined by crystal symmetry. An approximate (isotropic) treatment of cell e.s.d.'s is used for estimating e.s.d.'s involving l.s. planes.
Refinement. Refinement of *F*^2^ against ALL reflections. The weighted *R*-factor *wR* and goodness of fit *S* are based on *F*^2^, conventional *R*-factors *R* are based on *F*, with *F* set to zero for negative *F*^2^. The threshold expression of *F*^2^ > σ(*F*^2^) is used only for calculating *R*-factors(gt) *etc*. and is not relevant to the choice of reflections for refinement. *R*-factors based on *F*^2^ are statistically about twice as large as those based on *F*, and *R*- factors based on ALL data will be even larger.

### Fractional atomic coordinates and isotropic or equivalent isotropic displacement parameters (Å^2^)

**Table:**

	*x*	*y*	*z*	*U*_iso_*/*U*_eq_	
P1	0.22856 (4)	0.088527 (18)	0.29937 (3)	0.04277 (13)	
P2	0.08106 (4)	−0.000796 (19)	0.25127 (3)	0.04512 (13)	
N1	0.16074 (13)	0.04472 (7)	0.24696 (11)	0.0542 (4)	
B1	0.91160 (15)	0.33507 (8)	0.35287 (13)	0.0420 (4)	
C101	0.22876 (14)	0.14614 (7)	0.23395 (12)	0.0470 (4)	
C102	0.20818 (17)	0.14035 (9)	0.14671 (14)	0.0588 (5)	
H102	0.1933	0.1070	0.1230	0.071*	
C103	0.2096 (2)	0.18354 (11)	0.09540 (16)	0.0738 (7)	
H103	0.1961	0.1795	0.0369	0.089*	
C104	0.2308 (2)	0.23262 (10)	0.12998 (18)	0.0800 (7)	
H104	0.2305	0.2620	0.0948	0.096*	
C105	0.2527 (2)	0.23908 (9)	0.21653 (19)	0.0783 (7)	
H105	0.2682	0.2726	0.2395	0.094*	
C106	0.25171 (18)	0.19583 (8)	0.26909 (15)	0.0627 (6)	
H106	0.2663	0.2000	0.3276	0.075*	
C107	0.35801 (14)	0.06606 (8)	0.33088 (13)	0.0492 (4)	
C108	0.43941 (17)	0.10045 (10)	0.33679 (18)	0.0742 (7)	
H108	0.4286	0.1357	0.3204	0.089*	
C109	0.53677 (19)	0.08255 (13)	0.3670 (2)	0.0962 (10)	
H109	0.5915	0.1059	0.3709	0.115*	
C110	0.5536 (2)	0.03128 (14)	0.3912 (2)	0.0895 (9)	
H110	0.6197	0.0197	0.4118	0.107*	
C111	0.4741 (2)	−0.00317 (11)	0.38521 (19)	0.0833 (8)	
H111	0.4857	−0.0384	0.4016	0.100*	
C112	0.37625 (18)	0.01402 (9)	0.35500 (16)	0.0671 (6)	
H112	0.3221	−0.0097	0.3509	0.081*	
C113	0.18575 (14)	0.10953 (7)	0.39451 (12)	0.0455 (4)	
C114	0.23645 (16)	0.09499 (9)	0.47400 (13)	0.0578 (5)	
H114	0.2970	0.0758	0.4796	0.069*	
C115	0.1971 (2)	0.10899 (11)	0.54487 (15)	0.0724 (6)	
H115	0.2309	0.0989	0.5982	0.087*	
C116	0.1086 (2)	0.13773 (10)	0.53727 (16)	0.0740 (7)	
H116	0.0826	0.1471	0.5854	0.089*	
C117	0.05825 (19)	0.15274 (10)	0.45875 (16)	0.0704 (6)	
H117	−0.0017	0.1723	0.4538	0.084*	
C118	0.09619 (16)	0.13889 (8)	0.38735 (14)	0.0576 (5)	
H118	0.0619	0.1492	0.3342	0.069*	
C201	0.08078 (16)	−0.04373 (8)	0.16191 (13)	0.0536 (5)	
C202	−0.0057 (2)	−0.06931 (14)	0.12396 (19)	0.0982 (10)	
H202	−0.0675	−0.0625	0.1413	0.118*	
C203	−0.0013 (3)	−0.10510 (17)	0.0602 (2)	0.1279 (15)	
H203	−0.0601	−0.1231	0.0356	0.154*	
C204	0.0878 (3)	−0.11453 (14)	0.0326 (2)	0.1086 (11)	
H204	0.0896	−0.1382	−0.0115	0.130*	
C205	0.1749 (3)	−0.08897 (13)	0.0701 (2)	0.0983 (10)	
H205	0.2363	−0.0956	0.0519	0.118*	
C206	0.1715 (2)	−0.05368 (11)	0.13461 (18)	0.0802 (8)	
H206	0.2307	−0.0364	0.1601	0.096*	
C207	−0.04614 (15)	0.02379 (8)	0.24891 (12)	0.0494 (4)	
C208	−0.12093 (16)	−0.00698 (9)	0.27598 (14)	0.0591 (5)	
H208	−0.1053	−0.0410	0.2967	0.071*	
C209	−0.21786 (18)	0.01270 (12)	0.27225 (15)	0.0723 (7)	
H209	−0.2680	−0.0082	0.2895	0.087*	
C210	−0.2409 (2)	0.06321 (13)	0.24306 (18)	0.0826 (8)	
H210	−0.3061	0.0768	0.2421	0.099*	
C211	−0.1687 (2)	0.09353 (11)	0.2155 (2)	0.0900 (8)	
H211	−0.1851	0.1276	0.1951	0.108*	
C212	−0.07110 (18)	0.07410 (9)	0.21757 (17)	0.0709 (6)	
H212	−0.0224	0.0948	0.1979	0.085*	
C213	0.11038 (15)	−0.04065 (8)	0.34557 (13)	0.0500 (4)	
C214	0.09871 (16)	−0.01864 (9)	0.42253 (13)	0.0577 (5)	
H214	0.0696	0.0148	0.4241	0.069*	
C215	0.13004 (19)	−0.04620 (11)	0.49680 (15)	0.0719 (6)	
H215	0.1222	−0.0310	0.5482	0.086*	
C216	0.1718 (2)	−0.09477 (12)	0.49585 (19)	0.0883 (8)	
H216	0.1935	−0.1129	0.5463	0.106*	
C217	0.1820 (3)	−0.11706 (12)	0.4210 (2)	0.1101 (11)	
H217	0.2103	−0.1508	0.4205	0.132*	
C218	0.1510 (2)	−0.09061 (10)	0.34541 (18)	0.0825 (8)	
H218	0.1577	−0.1067	0.2945	0.099*	
C301	0.93347 (13)	0.31757 (7)	0.25836 (11)	0.0429 (4)	
C302	0.94568 (14)	0.26411 (7)	0.24073 (12)	0.0463 (4)	
C303	0.96279 (15)	0.24450 (8)	0.16489 (13)	0.0526 (5)	
C304	0.96834 (16)	0.27838 (9)	0.09941 (13)	0.0554 (5)	
C305	0.95460 (15)	0.33148 (9)	0.11172 (13)	0.0545 (5)	
C306	0.93588 (14)	0.34953 (7)	0.18875 (12)	0.0479 (4)	
C307	0.91300 (14)	0.39991 (7)	0.36903 (11)	0.0464 (4)	
C308	0.99731 (16)	0.42967 (8)	0.35719 (13)	0.0530 (5)	
C309	1.00838 (18)	0.48316 (9)	0.37307 (14)	0.0629 (6)	
C310	0.9343 (2)	0.50981 (8)	0.40492 (15)	0.0685 (6)	
C311	0.85109 (19)	0.48278 (9)	0.42072 (14)	0.0654 (6)	
C312	0.84172 (16)	0.42897 (8)	0.40271 (13)	0.0530 (5)	
C313	1.00187 (14)	0.31447 (7)	0.43143 (12)	0.0461 (4)	
C314	1.09755 (15)	0.29544 (8)	0.42482 (14)	0.0536 (5)	
C315	1.17082 (16)	0.28180 (9)	0.49416 (17)	0.0669 (6)	
C316	1.15124 (18)	0.28808 (10)	0.57383 (16)	0.0713 (7)	
C317	1.05887 (19)	0.30817 (10)	0.58441 (14)	0.0669 (6)	
C318	0.98807 (15)	0.32090 (8)	0.51411 (13)	0.0529 (5)	
C319	0.79839 (13)	0.30774 (7)	0.35379 (11)	0.0430 (4)	
C320	0.77678 (14)	0.26317 (7)	0.39764 (12)	0.0467 (4)	
C321	0.68163 (16)	0.24029 (8)	0.38989 (13)	0.0532 (5)	
C322	0.60085 (16)	0.26158 (9)	0.33584 (14)	0.0587 (5)	
C323	0.61746 (15)	0.30516 (9)	0.28948 (13)	0.0576 (5)	
C324	0.71371 (14)	0.32658 (8)	0.29868 (12)	0.0479 (4)	
F302	0.94100 (10)	0.22779 (4)	0.30197 (7)	0.0595 (3)	
F303	0.97370 (11)	0.19200 (5)	0.15422 (9)	0.0742 (4)	
F304	0.98522 (11)	0.26021 (6)	0.02472 (8)	0.0785 (4)	
F305	0.95914 (12)	0.36579 (5)	0.04829 (8)	0.0769 (4)	
F306	0.91801 (10)	0.40232 (4)	0.19218 (7)	0.0616 (3)	
F308	1.07512 (9)	0.40554 (5)	0.32848 (9)	0.0659 (3)	
F309	1.09217 (12)	0.50916 (6)	0.35857 (11)	0.0893 (5)	
F310	0.94318 (14)	0.56211 (5)	0.42144 (11)	0.1008 (5)	
F311	0.77742 (13)	0.50779 (6)	0.45293 (10)	0.0935 (5)	
F312	0.75641 (9)	0.40614 (5)	0.42110 (8)	0.0664 (3)	
F314	1.12606 (9)	0.28882 (6)	0.34921 (8)	0.0721 (4)	
F315	1.26140 (10)	0.26230 (7)	0.48210 (11)	0.0992 (5)	
F316	1.22159 (13)	0.27484 (8)	0.64166 (11)	0.1081 (6)	
F317	1.03870 (13)	0.31549 (8)	0.66281 (9)	0.1008 (5)	
F318	0.89929 (9)	0.34100 (6)	0.52933 (8)	0.0679 (3)	
F320	0.85042 (9)	0.23824 (4)	0.45267 (8)	0.0590 (3)	
F321	0.66743 (10)	0.19687 (5)	0.43540 (9)	0.0748 (4)	
F322	0.50761 (10)	0.23982 (7)	0.32793 (10)	0.0859 (4)	
F323	0.53981 (9)	0.32680 (7)	0.23466 (9)	0.0838 (4)	
F324	0.72415 (9)	0.36956 (5)	0.25024 (7)	0.0612 (3)	

### Atomic displacement parameters (Å^2^)

**Table:**

	*U*^11^	*U*^22^	*U*^33^	*U*^12^	*U*^13^	*U*^23^
P1	0.0420 (3)	0.0386 (2)	0.0477 (3)	−0.00422 (19)	0.0077 (2)	−0.00334 (19)
P2	0.0464 (3)	0.0417 (3)	0.0464 (3)	−0.0080 (2)	0.0059 (2)	−0.0044 (2)
N1	0.0582 (10)	0.0510 (9)	0.0530 (10)	−0.0141 (8)	0.0086 (8)	−0.0045 (7)
B1	0.0403 (11)	0.0400 (10)	0.0454 (11)	0.0034 (8)	0.0066 (9)	0.0007 (9)
C101	0.0431 (10)	0.0453 (10)	0.0545 (11)	−0.0031 (8)	0.0138 (8)	0.0000 (8)
C102	0.0622 (13)	0.0597 (13)	0.0559 (12)	−0.0098 (10)	0.0139 (10)	−0.0008 (10)
C103	0.0812 (17)	0.0812 (17)	0.0610 (14)	−0.0122 (13)	0.0184 (12)	0.0128 (12)
C104	0.0964 (19)	0.0672 (16)	0.0828 (18)	−0.0039 (14)	0.0331 (15)	0.0241 (14)
C105	0.103 (2)	0.0440 (12)	0.095 (2)	−0.0081 (12)	0.0368 (16)	0.0011 (12)
C106	0.0812 (15)	0.0469 (11)	0.0631 (13)	−0.0071 (10)	0.0213 (11)	−0.0029 (10)
C107	0.0462 (10)	0.0480 (11)	0.0541 (11)	0.0013 (8)	0.0106 (8)	−0.0097 (9)
C108	0.0492 (12)	0.0613 (14)	0.111 (2)	−0.0041 (10)	0.0112 (12)	−0.0058 (13)
C109	0.0464 (14)	0.097 (2)	0.143 (3)	−0.0048 (14)	0.0106 (15)	−0.0153 (19)
C110	0.0524 (15)	0.112 (2)	0.104 (2)	0.0246 (15)	0.0116 (14)	−0.0087 (18)
C111	0.0806 (18)	0.0742 (17)	0.095 (2)	0.0304 (15)	0.0159 (15)	0.0037 (14)
C112	0.0615 (13)	0.0541 (13)	0.0853 (16)	0.0069 (10)	0.0119 (12)	−0.0011 (11)
C113	0.0474 (10)	0.0404 (9)	0.0490 (10)	−0.0040 (8)	0.0096 (8)	−0.0015 (8)
C114	0.0520 (11)	0.0656 (13)	0.0533 (12)	0.0031 (10)	0.0023 (9)	−0.0056 (10)
C115	0.0772 (16)	0.0892 (17)	0.0493 (12)	0.0032 (13)	0.0070 (11)	−0.0033 (12)
C116	0.0854 (17)	0.0832 (17)	0.0589 (14)	0.0027 (14)	0.0280 (13)	−0.0085 (12)
C117	0.0711 (15)	0.0704 (15)	0.0755 (16)	0.0178 (12)	0.0290 (12)	0.0033 (12)
C118	0.0581 (12)	0.0584 (12)	0.0577 (12)	0.0119 (10)	0.0136 (10)	0.0076 (10)
C201	0.0562 (12)	0.0546 (12)	0.0504 (11)	−0.0132 (9)	0.0103 (9)	−0.0098 (9)
C202	0.0683 (16)	0.135 (3)	0.092 (2)	−0.0332 (17)	0.0164 (14)	−0.0606 (19)
C203	0.102 (2)	0.165 (4)	0.116 (3)	−0.048 (2)	0.017 (2)	−0.085 (3)
C204	0.131 (3)	0.116 (3)	0.083 (2)	−0.031 (2)	0.0339 (19)	−0.0561 (19)
C205	0.099 (2)	0.104 (2)	0.101 (2)	−0.0166 (18)	0.0425 (18)	−0.0427 (18)
C206	0.0694 (15)	0.0855 (17)	0.0899 (18)	−0.0198 (13)	0.0258 (13)	−0.0352 (15)
C207	0.0503 (11)	0.0502 (11)	0.0463 (10)	−0.0041 (9)	0.0042 (8)	−0.0026 (8)
C208	0.0541 (12)	0.0654 (13)	0.0574 (12)	−0.0054 (10)	0.0089 (10)	0.0011 (10)
C209	0.0535 (13)	0.100 (2)	0.0646 (14)	−0.0073 (13)	0.0125 (11)	−0.0033 (13)
C210	0.0543 (14)	0.103 (2)	0.0866 (18)	0.0136 (14)	0.0031 (13)	−0.0163 (16)
C211	0.0741 (18)	0.0708 (17)	0.117 (2)	0.0143 (14)	−0.0046 (16)	0.0090 (16)
C212	0.0595 (14)	0.0594 (14)	0.0907 (17)	0.0003 (11)	0.0042 (12)	0.0106 (12)
C213	0.0479 (10)	0.0448 (10)	0.0557 (12)	−0.0067 (8)	0.0050 (9)	0.0008 (9)
C214	0.0595 (12)	0.0587 (12)	0.0546 (12)	−0.0034 (10)	0.0089 (10)	0.0011 (10)
C215	0.0724 (15)	0.0866 (18)	0.0551 (13)	−0.0064 (13)	0.0069 (11)	0.0059 (12)
C216	0.098 (2)	0.087 (2)	0.0738 (18)	0.0075 (16)	0.0001 (15)	0.0252 (15)
C217	0.159 (3)	0.0665 (17)	0.101 (2)	0.0421 (19)	0.013 (2)	0.0207 (17)
C218	0.116 (2)	0.0552 (14)	0.0746 (17)	0.0185 (14)	0.0122 (15)	0.0001 (12)
C301	0.0374 (9)	0.0428 (10)	0.0478 (10)	0.0004 (7)	0.0055 (8)	−0.0002 (8)
C302	0.0449 (10)	0.0439 (10)	0.0498 (11)	0.0006 (8)	0.0073 (8)	0.0016 (8)
C303	0.0513 (11)	0.0456 (11)	0.0611 (12)	−0.0009 (8)	0.0103 (9)	−0.0108 (9)
C304	0.0545 (12)	0.0646 (13)	0.0482 (11)	−0.0031 (10)	0.0121 (9)	−0.0122 (10)
C305	0.0552 (12)	0.0616 (13)	0.0473 (11)	−0.0041 (10)	0.0113 (9)	0.0062 (9)
C306	0.0487 (10)	0.0419 (10)	0.0531 (11)	−0.0010 (8)	0.0085 (8)	0.0009 (8)
C307	0.0486 (10)	0.0446 (10)	0.0435 (10)	0.0048 (8)	0.0012 (8)	−0.0018 (8)
C308	0.0547 (12)	0.0484 (11)	0.0528 (11)	−0.0012 (9)	0.0008 (9)	−0.0032 (9)
C309	0.0701 (14)	0.0510 (12)	0.0612 (13)	−0.0111 (11)	−0.0061 (11)	−0.0009 (10)
C310	0.0911 (18)	0.0402 (11)	0.0653 (14)	0.0018 (11)	−0.0111 (12)	−0.0093 (10)
C311	0.0793 (16)	0.0544 (13)	0.0573 (13)	0.0225 (12)	−0.0022 (11)	−0.0126 (10)
C312	0.0551 (12)	0.0521 (11)	0.0494 (11)	0.0085 (9)	0.0025 (9)	−0.0031 (9)
C313	0.0424 (10)	0.0415 (10)	0.0522 (11)	0.0000 (8)	0.0025 (8)	0.0016 (8)
C314	0.0456 (11)	0.0520 (11)	0.0609 (12)	0.0030 (9)	0.0033 (9)	−0.0023 (9)
C315	0.0439 (12)	0.0655 (14)	0.0854 (17)	0.0069 (10)	−0.0053 (11)	0.0032 (12)
C316	0.0595 (14)	0.0737 (15)	0.0698 (16)	0.0021 (11)	−0.0189 (12)	0.0110 (12)
C317	0.0733 (15)	0.0719 (15)	0.0501 (13)	−0.0051 (12)	−0.0042 (11)	0.0057 (11)
C318	0.0486 (11)	0.0543 (12)	0.0541 (12)	0.0004 (9)	0.0040 (9)	0.0018 (9)
C319	0.0417 (10)	0.0455 (10)	0.0417 (10)	0.0038 (8)	0.0073 (8)	−0.0026 (8)
C320	0.0472 (10)	0.0464 (10)	0.0458 (10)	0.0043 (8)	0.0063 (8)	0.0003 (8)
C321	0.0573 (12)	0.0499 (11)	0.0534 (11)	−0.0073 (9)	0.0126 (9)	−0.0006 (9)
C322	0.0455 (11)	0.0729 (14)	0.0571 (12)	−0.0124 (10)	0.0075 (9)	−0.0053 (11)
C323	0.0427 (11)	0.0766 (14)	0.0501 (11)	0.0060 (10)	−0.0016 (9)	0.0001 (10)
C324	0.0473 (11)	0.0510 (11)	0.0450 (10)	0.0036 (8)	0.0072 (8)	0.0034 (8)
F302	0.0773 (8)	0.0417 (6)	0.0612 (7)	0.0070 (5)	0.0170 (6)	0.0067 (5)
F303	0.0954 (10)	0.0495 (7)	0.0810 (9)	0.0035 (6)	0.0244 (7)	−0.0173 (6)
F304	0.0904 (10)	0.0924 (10)	0.0574 (8)	−0.0034 (8)	0.0261 (7)	−0.0191 (7)
F305	0.1014 (10)	0.0772 (9)	0.0558 (7)	−0.0018 (7)	0.0238 (7)	0.0140 (6)
F306	0.0833 (8)	0.0429 (6)	0.0588 (7)	0.0033 (5)	0.0135 (6)	0.0070 (5)
F308	0.0517 (7)	0.0638 (7)	0.0835 (9)	−0.0074 (6)	0.0159 (6)	−0.0063 (6)
F309	0.0929 (11)	0.0649 (8)	0.1046 (11)	−0.0309 (8)	0.0017 (8)	0.0006 (8)
F310	0.1391 (15)	0.0436 (7)	0.1065 (12)	0.0027 (8)	−0.0149 (10)	−0.0187 (7)
F311	0.1077 (12)	0.0761 (9)	0.0955 (11)	0.0365 (8)	0.0145 (9)	−0.0252 (8)
F312	0.0588 (7)	0.0692 (8)	0.0741 (8)	0.0123 (6)	0.0193 (6)	−0.0087 (6)
F314	0.0485 (7)	0.0950 (10)	0.0732 (8)	0.0164 (6)	0.0115 (6)	−0.0094 (7)
F315	0.0496 (8)	0.1182 (13)	0.1218 (13)	0.0288 (8)	−0.0072 (8)	0.0010 (10)
F316	0.0850 (11)	0.1322 (14)	0.0890 (11)	0.0130 (10)	−0.0353 (9)	0.0211 (10)
F317	0.1074 (12)	0.1400 (15)	0.0498 (8)	0.0071 (11)	−0.0006 (8)	0.0090 (8)
F318	0.0613 (7)	0.0901 (9)	0.0535 (7)	0.0097 (6)	0.0135 (6)	−0.0017 (6)
F320	0.0547 (7)	0.0544 (7)	0.0653 (7)	0.0043 (5)	0.0033 (5)	0.0157 (5)
F321	0.0767 (9)	0.0646 (8)	0.0830 (9)	−0.0181 (6)	0.0140 (7)	0.0145 (7)
F322	0.0519 (7)	0.1139 (12)	0.0890 (10)	−0.0278 (7)	0.0045 (7)	0.0040 (9)
F323	0.0474 (7)	0.1192 (12)	0.0777 (9)	0.0048 (7)	−0.0092 (6)	0.0232 (8)
F324	0.0561 (7)	0.0659 (7)	0.0585 (7)	0.0054 (6)	0.0018 (5)	0.0185 (6)

### Geometric parameters (Å, º)

**Table:**

P1—N1	1.5727 (16)	C209—H209	0.9300
P1—C101	1.7913 (19)	C210—C211	1.361 (4)
P1—C113	1.7992 (19)	C210—H210	0.9300
P1—C107	1.800 (2)	C211—C212	1.383 (4)
P2—N1	1.5717 (16)	C211—H211	0.9300
P2—C201	1.795 (2)	C212—H212	0.9300
P2—C207	1.797 (2)	C213—C218	1.370 (3)
P2—C213	1.800 (2)	C213—C214	1.386 (3)
B1—C301	1.653 (3)	C214—C215	1.378 (3)
B1—C307	1.654 (3)	C214—H214	0.9300
B1—C319	1.659 (3)	C215—C216	1.346 (4)
B1—C313	1.664 (3)	C215—H215	0.9300
C101—C106	1.385 (3)	C216—C217	1.352 (4)
C101—C102	1.385 (3)	C216—H216	0.9300
C102—C103	1.367 (3)	C217—C218	1.382 (4)
C102—H102	0.9300	C217—H217	0.9300
C103—C104	1.364 (4)	C218—H218	0.9300
C103—H103	0.9300	C301—C306	1.381 (3)
C104—C105	1.377 (4)	C301—C302	1.392 (3)
C104—H104	0.9300	C302—F302	1.352 (2)
C105—C106	1.379 (3)	C302—C303	1.368 (3)
C105—H105	0.9300	C303—F303	1.345 (2)
C106—H106	0.9300	C303—C304	1.365 (3)
C107—C112	1.376 (3)	C304—F304	1.338 (2)
C107—C108	1.377 (3)	C304—C305	1.369 (3)
C108—C109	1.377 (4)	C305—F305	1.344 (2)
C108—H108	0.9300	C305—C306	1.379 (3)
C109—C110	1.356 (4)	C306—F306	1.354 (2)
C109—H109	0.9300	C307—C312	1.381 (3)
C110—C111	1.360 (4)	C307—C308	1.390 (3)
C110—H110	0.9300	C308—F308	1.349 (2)
C111—C112	1.377 (3)	C308—C309	1.375 (3)
C111—H111	0.9300	C309—F309	1.348 (3)
C112—H112	0.9300	C309—C310	1.363 (3)
C113—C114	1.383 (3)	C310—F310	1.345 (2)
C113—C118	1.390 (3)	C310—C311	1.362 (4)
C114—C115	1.378 (3)	C311—F311	1.343 (3)
C114—H114	0.9300	C311—C312	1.387 (3)
C115—C116	1.369 (4)	C312—F312	1.351 (2)
C115—H115	0.9300	C313—C318	1.380 (3)
C116—C117	1.372 (4)	C313—C314	1.382 (3)
C116—H116	0.9300	C314—F314	1.343 (2)
C117—C118	1.375 (3)	C314—C315	1.387 (3)
C117—H117	0.9300	C315—F315	1.347 (3)
C118—H118	0.9300	C315—C316	1.357 (4)
C201—C202	1.365 (3)	C316—F316	1.347 (3)
C201—C206	1.378 (3)	C316—C317	1.367 (3)
C202—C203	1.372 (4)	C317—F317	1.343 (3)
C202—H202	0.9300	C317—C318	1.374 (3)
C203—C204	1.357 (5)	C318—F318	1.346 (2)
C203—H203	0.9300	C319—C320	1.382 (3)
C204—C205	1.370 (4)	C319—C324	1.388 (3)
C204—H204	0.9300	C320—F320	1.353 (2)
C205—C206	1.371 (4)	C320—C321	1.377 (3)
C205—H205	0.9300	C321—F321	1.347 (2)
C206—H206	0.9300	C321—C322	1.367 (3)
C207—C212	1.383 (3)	C322—F322	1.342 (2)
C207—C208	1.390 (3)	C322—C323	1.366 (3)
C208—C209	1.374 (3)	C323—F323	1.349 (2)
C208—H208	0.9300	C323—C324	1.374 (3)
C209—C210	1.372 (4)	C324—F324	1.354 (2)
			
N1—P1—C101	108.38 (9)	C211—C210—C209	120.2 (2)
N1—P1—C113	114.97 (9)	C211—C210—H210	119.9
C101—P1—C113	106.94 (9)	C209—C210—H210	119.9
N1—P1—C107	111.15 (9)	C210—C211—C212	120.5 (3)
C101—P1—C107	108.65 (9)	C210—C211—H211	119.8
C113—P1—C107	106.52 (9)	C212—C211—H211	119.8
N1—P2—C201	108.16 (9)	C207—C212—C211	119.9 (2)
N1—P2—C207	112.82 (9)	C207—C212—H212	120.1
C201—P2—C207	108.72 (9)	C211—C212—H212	120.1
N1—P2—C213	113.29 (9)	C218—C213—C214	118.4 (2)
C201—P2—C213	107.71 (10)	C218—C213—P2	122.58 (18)
C207—P2—C213	105.94 (9)	C214—C213—P2	118.88 (16)
P2—N1—P1	144.79 (12)	C215—C214—C213	120.2 (2)
C301—B1—C307	114.10 (15)	C215—C214—H214	119.9
C301—B1—C319	101.75 (14)	C213—C214—H214	119.9
C307—B1—C319	113.19 (15)	C216—C215—C214	120.8 (2)
C301—B1—C313	113.04 (15)	C216—C215—H215	119.6
C307—B1—C313	101.69 (14)	C214—C215—H215	119.6
C319—B1—C313	113.59 (15)	C215—C216—C217	119.6 (3)
C106—C101—C102	119.78 (19)	C215—C216—H216	120.2
C106—C101—P1	121.14 (16)	C217—C216—H216	120.2
C102—C101—P1	119.06 (15)	C216—C217—C218	121.2 (3)
C103—C102—C101	120.2 (2)	C216—C217—H217	119.4
C103—C102—H102	119.9	C218—C217—H217	119.4
C101—C102—H102	119.9	C213—C218—C217	119.9 (3)
C104—C103—C102	120.1 (2)	C213—C218—H218	120.1
C104—C103—H103	120.0	C217—C218—H218	120.1
C102—C103—H103	120.0	C306—C301—C302	112.34 (17)
C103—C104—C105	120.6 (2)	C306—C301—B1	128.20 (16)
C103—C104—H104	119.7	C302—C301—B1	119.35 (16)
C105—C104—H104	119.7	F302—C302—C303	115.92 (16)
C104—C105—C106	120.0 (2)	F302—C302—C301	119.07 (16)
C104—C105—H105	120.0	C303—C302—C301	125.00 (18)
C106—C105—H105	120.0	F303—C303—C304	119.71 (18)
C105—C106—C101	119.4 (2)	F303—C303—C302	120.45 (19)
C105—C106—H106	120.3	C304—C303—C302	119.84 (18)
C101—C106—H106	120.3	F304—C304—C303	120.99 (19)
C112—C107—C108	118.8 (2)	F304—C304—C305	120.7 (2)
C112—C107—P1	119.44 (16)	C303—C304—C305	118.27 (18)
C108—C107—P1	121.60 (17)	F305—C305—C304	119.63 (19)
C109—C108—C107	119.9 (2)	F305—C305—C306	120.30 (19)
C109—C108—H108	120.1	C304—C305—C306	120.06 (18)
C107—C108—H108	120.1	F306—C306—C305	114.89 (17)
C110—C109—C108	120.7 (3)	F306—C306—C301	120.71 (17)
C110—C109—H109	119.6	C305—C306—C301	124.39 (18)
C108—C109—H109	119.6	C312—C307—C308	113.18 (18)
C109—C110—C111	120.0 (2)	C312—C307—B1	126.54 (18)
C109—C110—H110	120.0	C308—C307—B1	119.91 (16)
C111—C110—H110	120.0	F308—C308—C309	116.18 (19)
C110—C111—C112	120.0 (3)	F308—C308—C307	119.32 (17)
C110—C111—H111	120.0	C309—C308—C307	124.5 (2)
C112—C111—H111	120.0	F309—C309—C310	120.0 (2)
C107—C112—C111	120.5 (2)	F309—C309—C308	120.6 (2)
C107—C112—H112	119.7	C310—C309—C308	119.4 (2)
C111—C112—H112	119.7	F310—C310—C311	119.9 (2)
C114—C113—C118	119.32 (18)	F310—C310—C309	120.8 (2)
C114—C113—P1	121.85 (15)	C311—C310—C309	119.2 (2)
C118—C113—P1	118.74 (15)	F311—C311—C310	120.6 (2)
C115—C114—C113	119.9 (2)	F311—C311—C312	119.6 (2)
C115—C114—H114	120.1	C310—C311—C312	119.8 (2)
C113—C114—H114	120.1	F312—C312—C307	121.39 (18)
C116—C115—C114	120.5 (2)	F312—C312—C311	114.77 (19)
C116—C115—H115	119.8	C307—C312—C311	123.8 (2)
C114—C115—H115	119.8	C318—C313—C314	113.21 (17)
C115—C116—C117	120.1 (2)	C318—C313—B1	119.40 (16)
C115—C116—H116	119.9	C314—C313—B1	127.07 (17)
C117—C116—H116	119.9	F314—C314—C313	121.49 (18)
C116—C117—C118	120.2 (2)	F314—C314—C315	115.01 (19)
C116—C117—H117	119.9	C313—C314—C315	123.5 (2)
C118—C117—H117	119.9	F315—C315—C316	120.1 (2)
C117—C118—C113	120.1 (2)	F315—C315—C314	119.7 (2)
C117—C118—H118	120.0	C316—C315—C314	120.1 (2)
C113—C118—H118	120.0	F316—C316—C315	120.7 (2)
C202—C201—C206	119.3 (2)	F316—C316—C317	120.3 (2)
C202—C201—P2	121.60 (18)	C315—C316—C317	119.0 (2)
C206—C201—P2	118.94 (16)	F317—C317—C316	119.9 (2)
C201—C202—C203	120.0 (3)	F317—C317—C318	121.0 (2)
C201—C202—H202	120.0	C316—C317—C318	119.1 (2)
C203—C202—H202	120.0	F318—C318—C317	115.86 (19)
C204—C203—C202	120.8 (3)	F318—C318—C313	119.16 (17)
C204—C203—H203	119.6	C317—C318—C313	125.0 (2)
C202—C203—H203	119.6	C320—C319—C324	112.57 (17)
C203—C204—C205	119.7 (3)	C320—C319—B1	127.74 (16)
C203—C204—H204	120.1	C324—C319—B1	119.32 (16)
C205—C204—H204	120.1	F320—C320—C321	114.46 (17)
C206—C205—C204	119.9 (3)	F320—C320—C319	121.18 (16)
C206—C205—H205	120.1	C321—C320—C319	124.36 (18)
C204—C205—H205	120.1	F321—C321—C322	119.54 (18)
C205—C206—C201	120.3 (2)	F321—C321—C320	120.35 (18)
C205—C206—H206	119.8	C322—C321—C320	120.11 (19)
C201—C206—H206	119.8	F322—C322—C323	120.8 (2)
C212—C207—C208	119.0 (2)	F322—C322—C321	120.7 (2)
C212—C207—P2	119.29 (16)	C323—C322—C321	118.45 (19)
C208—C207—P2	121.65 (16)	F323—C323—C322	120.07 (19)
C209—C208—C207	120.2 (2)	F323—C323—C324	120.3 (2)
C209—C208—H208	119.9	C322—C323—C324	119.63 (18)
C207—C208—H208	119.9	F324—C324—C323	116.12 (17)
C210—C209—C208	120.1 (2)	F324—C324—C319	119.03 (16)
C210—C209—H209	119.9	C323—C324—C319	124.86 (18)
C208—C209—H209	119.9		

### Hydrogen-bond geometry (Å, º)

**Table:**

*D*—H···*A*	*D*—H	H···*A*	*D*···*A*	*D*—H···*A*
C118—H118···F302^i^	0.93	2.55	3.188 (2)	126
C212—H212···F303^i^	0.93	2.55	3.229 (3)	131

Symmetry code: (i) *x*−1, *y*, *z*.
